# An Efficient Method for Deidentifying Protected Health Information in Chinese Electronic Health Records: Algorithm Development and Validation

**DOI:** 10.2196/38154

**Published:** 2022-08-30

**Authors:** Peng Wang, Yong Li, Liang Yang, Simin Li, Linfeng Li, Zehan Zhao, Shaopei Long, Fei Wang, Hongqian Wang, Ying Li, Chengliang Wang

**Affiliations:** 1 College of Computer Science Chongqing University Chongqing China; 2 School of Computer Science South China Normal University Guangzhou China; 3 Yidu Cloud Technology Inc Beijing China; 4 School of Software & Microelectronics Peking University Beijing China; 5 Medical Big Data Center of Southwest Hospital Chongqing China

**Keywords:** EHR, PHI, personal information, protected data, protected information, patient information, health information, de-identification, de-identify, privacy, TinyBert, model, development, algorithm, machine learning, CRF, data augmentation, health record, medical record

## Abstract

**Background:**

With the popularization of electronic health records in China, the utilization of digitalized data has great potential for the development of real-world medical research. However, the data usually contains a great deal of protected health information and the direct usage of this data may cause privacy issues. The task of deidentifying protected health information in electronic health records can be regarded as a named entity recognition problem. Existing rule-based, machine learning–based, or deep learning–based methods have been proposed to solve this problem. However, these methods still face the difficulties of insufficient Chinese electronic health record data and the complex features of the Chinese language.

**Objective:**

This paper proposes a method to overcome the difficulties of overfitting and a lack of training data for deep neural networks to enable Chinese protected health information deidentification.

**Methods:**

We propose a new model that merges TinyBERT (bidirectional encoder representations from transformers) as a text feature extraction module and the conditional random field method as a prediction module for deidentifying protected health information in Chinese medical electronic health records. In addition, a hybrid data augmentation method that integrates a sentence generation strategy and a mention-replacement strategy is proposed for overcoming insufficient Chinese electronic health records.

**Results:**

We compare our method with 5 baseline methods that utilize different BERT models as their feature extraction modules. Experimental results on the Chinese electronic health records that we collected demonstrate that our method had better performance (microprecision: 98.7%, microrecall: 99.13%, and micro-F1 score: 98.91%) and higher efficiency (40% faster) than all the BERT-based baseline methods.

**Conclusions:**

Compared to baseline methods, the efficiency advantage of TinyBERT on our proposed augmented data set was kept while the performance improved for the task of Chinese protected health information deidentification.

## Introduction

### Background

With the boost in information technology, electronic health records (EHRs) have been widely adopted and applied in many hospitals and medical institutes. The vast advantages of EHRs include easy storage and management, and they can greatly increase the speed of information retrieval. They can provide abundant clinical and medical information on various diseases, and this information can potentially provide clinicians with evidence for decision-making. However, the private information of many individuals is stored in the EHRs. The incorrect usage of EHRs may cause privacy leakage, leading to serious problems. In order to standardize the use of EHRs and protect individual privacy, many projects, such as the i2b2 challenge, in 2014 [1], and the CEGS N-GRID challenge, in 2016 [2], have been launched. An intuitive method to prevent privacy leakage is deidentifying the protected health information (PHI) [3] in EHRs before information processing. PHI is classified into 18 different types by the US Health Insurance Portability and Accountability Act [4], such as name, ID number, location, date, and age. The process of deidentifying PHI can be divided into 2 steps: locating the PHI in the EHR and replacing it with information that is not sensitive. Accordingly, the deidentification procedure can be treated as a named entity recognition (NER) task [5].

### Related Work

In the past few decades, rule-based [6,7] and machine learning–based [3,8,9] approaches have been the mainstream approaches to identifying entities in sentences or documents. Rule-based methods utilize special semantic dictionaries to establish a set of regular expressions [4,5] to extract PHI from EHRs. However, these methods are labor intensive and time consuming, with poor generalization capability. Machine learning methods based on the principles of statistics could automatically detect PHI in EHRs by utilizing manually extracted text features [3,10]. For example, Jian et al [11] designed a set of regular expressions based on the characteristics of Chinese EHRs to filter sentences with sparse PHI, then used the filtered sentences to train a conditional random field (CRF) model for PHI recognition. Du et al [12] manually extracted lexical and dictionary features of PHI from Chinese EHRs to train a CRF model and utilized regular expressions to capture missed features using the CRF. On the basis of the extracted lexical features, Zhang et al [13] employed a long short-term memory (LSTM) method to learn the features of PHI sentences. However, these machine learning–based methods heavily depend on high-quality manual selection of features, which requires a great amount of domain expertise. In recent years, many deep learning models have been applied to the deidentification of PHI. Compared to rule-based and machine learning–based methods, deep learning models could extract features automatically from input words or text vectors [14,15]. However, deep learning–based models require very large annotated data sets for model training to avoid overfitting. To solve this problem, it is tempting to perform data augmentation [16,17] when facing data set insufficiency.

Currently, deidentifying PHI with deep neural networks remains a greater challenge for Chinese-language clinical texts than for other languages [18]. At present, much existing research on PHI deidentification has been done on the English-language corpus. Increasing performance has been achieved for rule-based, machine learning–based, deep learning–based, and hybrid approaches [19,20]. However, the direct application of these methods to Chinese clinical texts for PHI deidentification may result in unsatisfactory results. The huge differences in morphological features between Chinese and English make it futile to construct rules and dictionaries. For example, there is no delimiter in the middle of a sentence in Chinese, because the basic morpheme that expresses meaning in Chinese consists of more than one word. Additionally, Chinese grammar is more flexible, and some words can exist as multiple parts of speech. In addition, the absence of capitalization makes it difficult to locate personal names in Chinese through specific rules. As a result, deep neural networks require a very large Chinese biomedical corpus for learning the high level contextual semantic features of Chinese. However, annotating a large amount of Chinese data for network training is costly, labor intensive, and time consuming. Thus, there is a great need for the ability to train deep neural networks on limited-size annotated Chinese data sets. To reduce model dependence on limited training data, an intuitive method would be to fine-tune a model that has been pretrained with a Chinese corpus with the target-specific downstream data set. However, there are two limitations on applying pretrained language models to downstream tasks. First, if the pretraining tasks and the target tasks are not domain matched, the pretraining model may impair the performance of the target tasks [21]. Second, there can be overfitting issues when there is not enough data for fine tuning the downstream tasks.

### Objective

In this paper, we propose a deep neural network that uses TinyBERT [22] and a CRF model for Chinese PHI deidentification. TinyBERT as used in our model is distilled from a BERT (bidirectional encoder representations from transformers)-based model that was pretrained on a Chinese corpus. It has two advantages: it can overcome the differences in the morphological features of Chinese and English, and it has fewer parameters, which should prevent the deep learning model from overfitting when training on small-scale Chinese EHR data sets. In addition, we propose a hybrid data-augmentation method that uses data augmentation with a generation approach (DAGA) [23] and mention replacement (MR) [24] to create more training data. The enhanced data set assists the neural network in overcoming overfitting and enhances the generalizability of the deep neural networks.

## Methods

### The PHI Recognition Model

**Figure 1:**
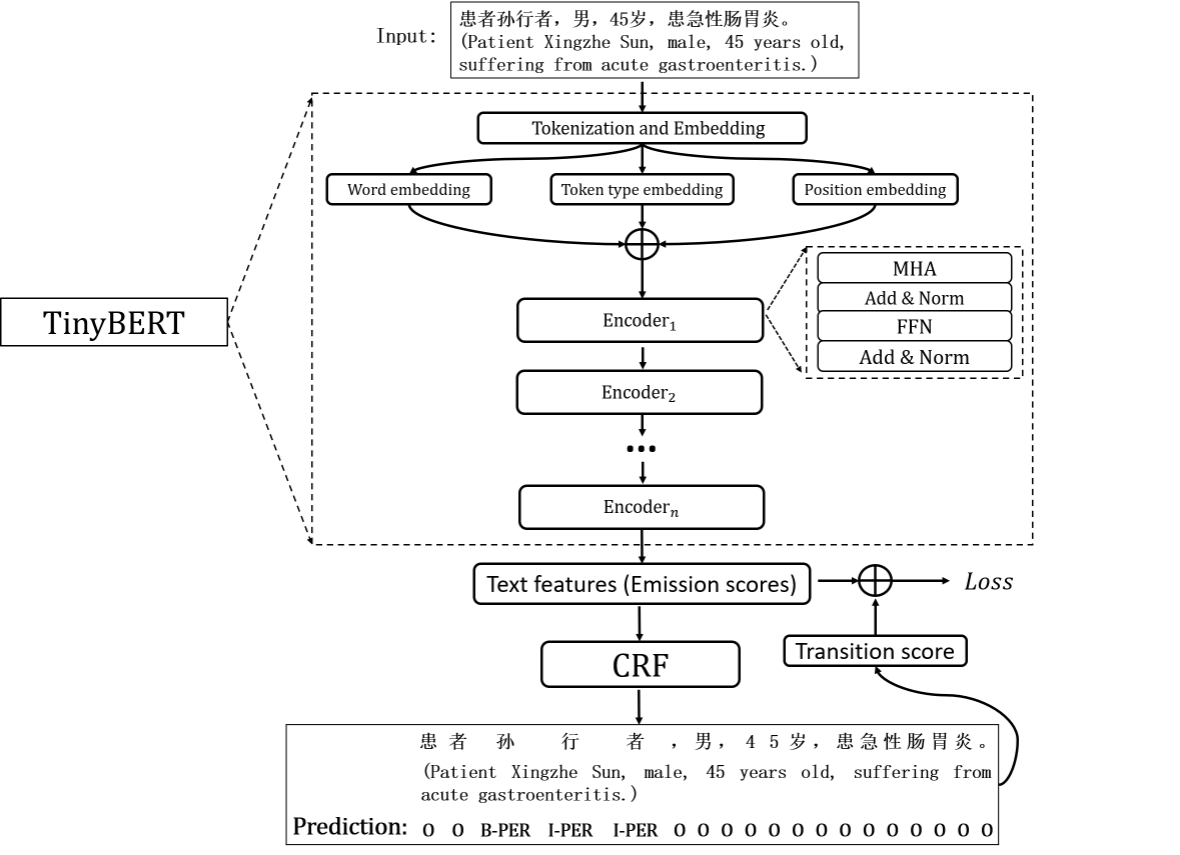
The proposed model for deidentifying protected health information in Chinese electronic health records. BERT: bidirectional encoder representations from transformers; CRF: conditional random field; FFN: feed-forward network; MHA: multi-head attention; PER: personal name.

In this paper, a new model that integrates TinyBERT [22] and a CRF model [25] is proposed for PHI recognition in Chinese EHRs. As shown in Figure 1, this model utilizes TinyBERT as the feature extraction module and the CRF model as the prediction module. The words in the sentences of an EHR are first tokenized, and the lengths of the sentences are fixed to 128. They are then input to the embedding module of TinyBERT to generate word embeddings, position embeddings, and token-type embeddings. The 3 embedding matrices are added together as input to the feature encoder, consisting of cascaded self-attention blocks for text feature extraction. With the self-attention mechanism, the model captures long-distance interdependent features in sentences and learns the semantics of the sentences. The feature extraction module outputs a series of probabilities for sequence labels, which are regarded as the emission scores of the CRF model. After that, the text features are input to the CRF module for label prediction.

**Figure 2:**
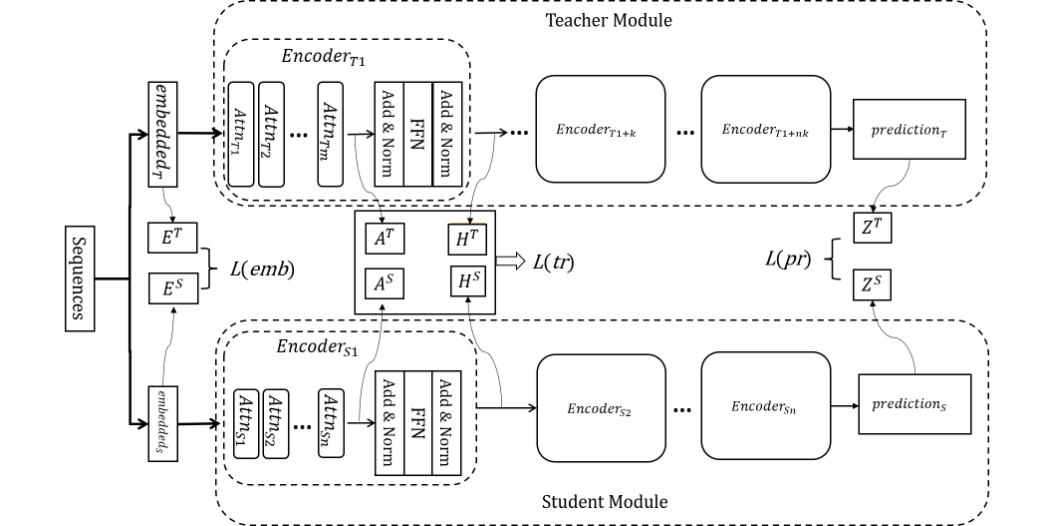
The TinyBERT knowledge distillation process used in our model. FFN: feed-forward network. Attn: attention layer; L(emb): embedding loss; L(tr): transformer layer loss; L(pr): prediction loss; A: attention map values; Z: predicted logic vectors; S: student network; T: teacher network.

TinyBERT is a light structure which is generated with the transformer-layer distillation method from the base BERT [26]. The structures to be distilled are an embedding layer, multiple transformer layers, and a prediction layer. The details of the model distillation process are shown in Figure 2. Assuming that the base BERT is the teacher module and has M transformer layers, TinyBERT is the student module and has N transformer layers, where M = k × N. In the distillation process, the model learns knowledge through a knowledge distillation (KD) function between the indices from the teacher module to student module, as shown in equation 1:


θ_S_(n) = g(k,θ_T_(m)) **(1)**

where θ_S_(n) denotes the parameters of the student module with n transformer layers, θ_T_(m) denotes the parameters of the teacher module with m transformer layers, and g(•) denotes the knowledge mapping function from the teacher module to the student module. Formally, g(•) is optimized through minimizing the distillation loss (L(distillation)), which is summed by the transformer layer loss (L(tr)), the embedding layer loss (L(emb)), and the prediction loss (L(pr)). To generate the TinyBERT model, training sequences with a length of 1 were simultaneously input to the teacher module and the student module for label prediction, and the distillation loss was then minimized in the training process, which can be calculated from equation 2 to equation 5, as follows:



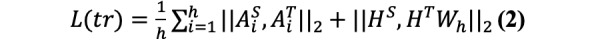




L(emb) = ||E^S^,E^T^W_e_||_2_
**(3)**

L(pr) = cross_entropy (Z^T^,Z^S^) **(4)**

L(distillation) = L(tr) + L(emb) + L(pr) **(5)**

where h is the number of attention heads. 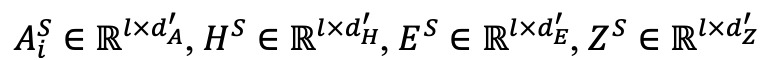 denote the *i-th-*layer attention map values, the output feature maps of the transformer blocks, the output of the embedding layers, and the predicted logic vectors of the student module, respectively. 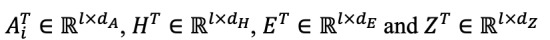 denote the *i-th-*layer attention map values, the output feature maps of the transformer blocks, the output of the embedding layer, and the predicted logic vectors of the teacher module, respectively. W_h_ and W_e_ denote the linear transformation matrices, and 
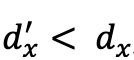
, where 𝑥∈{A, H,E,Z}.

After the knowledge distillation process, the parameters of the obtained TinyBERT were dramatically shrunk, while reserving most of the knowledge of the base BERT. Our model utilizes the text features output by the last TinyBERT encoder to finally obtain the predicted labels through a classifier, such as a softmax function. However, the softmax function regards each vector as independent and ignores correlations between word labels in a sentence; thus, some unreasonable results may be predicted. To eliminate this issue, we introduced the CRF model to build the dependencies and constraints within annotated sequences. Instead of assuming that the current label of a token depends only on the current label or the current label depends only on a previous label, the CRF model breaks the limitations of local token dependencies and focuses on the whole sentence. Specific dependency rules that can be learned in the NER task are shown in Figure 2.

The label for the first word in a sentence should start with “B-” or “O,” not “I-.” In the mode that “B - label_1 I - label_2 I - label_3 I -...” there should be the same named entity tag for label_1, label_2, and label_3. Based on this rule, it is easy to exclude wrong predictions, such as “B-Person I-Organization...” Based on the observations, the CRF model can define an equation to score a predicted sequence label of the input sentence according to the dependency rules in equations (6) to (8):


score(X|s) = emission_score + transition_score **(6)**

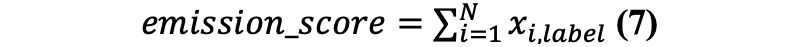





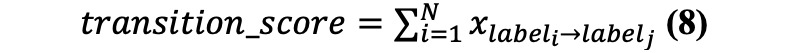



where s denotes the input sentence, 𝑥_i,label_ denotes the score of the predicted labels of the *i-th* word in the sentence s, and 𝑥_labeli→labelj_ denotes the score of transferring the *label_i_* to *label_j_* of the word 𝑥, respectively. In our method, the *emission_score* is obtained from the output of the TinyBERT module, and the *transition_score* is calculated by the CRF module with the contextual information in the sentence. To maximize the probability of correct predicted sequence labels, the exponent and standardization among all the predicted scores are calculated according to equation 9:



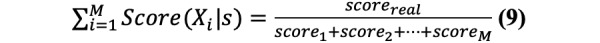



Therefore, the loss function for optimizing our model can be defined as equation 10:



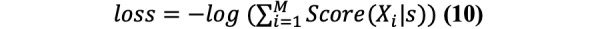



### A Hybrid Data Augmentation Method

Formally, there is a trade-off between the performance and efficiency of a deep neural network. The performance of a network degrades, while its efficiency is enhanced, when the parameters are compressed. In practice, a network compresses the number of transformer layers and word embedding dimensions to improve efficiency, but this also results in the ability of feature extraction becoming inferior. To keep its efficiency without degrading its performance, an intuitive method is to fine-tune it on a large data set. Unfortunately, the generation of a sufficient, high-quality data set is challenging. As discussed in previous reports [23,24], augmenting data with noise may enhance the robustness of the models on tasks at the sentence level, such as text classification and emotional judgment, but it harms the performance of tasks at the token level, such as NER. This situation indicates that augmented data should contain as little noise as possible. Furthermore, the research of Dai et al [27] indicates that hybrid data augmentation outperforms any single method of data augmentation, on average. Inspired by this work, we propose a new hybrid data augmentation method, which combines DAGA [23] and MR [24] to enhance original data for task-specific fine-tuning. The DAGA is used to increase the size of the training set so as to avoid overfitting, while MR is used to enable a network to learn different representations of entities.

**Figure 3:**
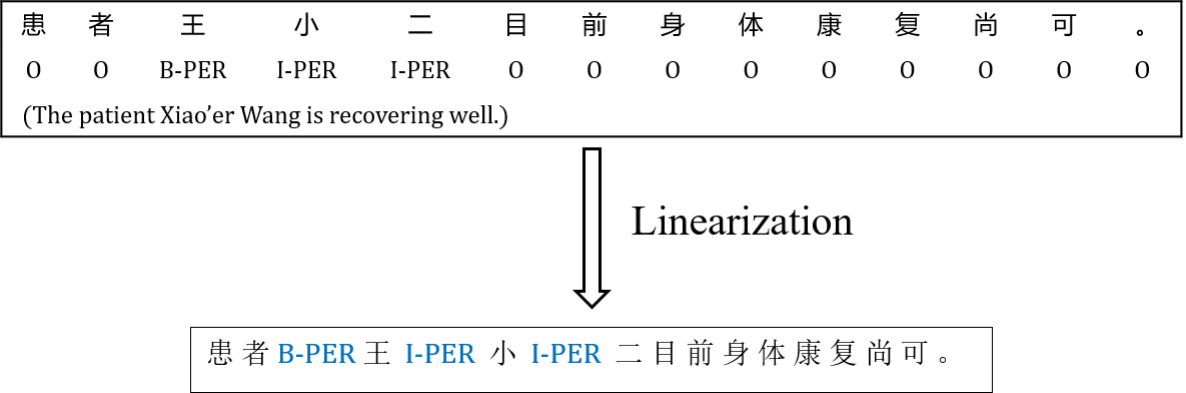
An example of the data augmentation with a generation approach linearization operation in our data augmentation method. PER: personal name.

Unlike other data augmentation methods, a DAGA generates new synthetic data from scratch without relying on WordNet (Princeton University) or other external dictionaries, which could make it more useful for limited-resource languages. It mixes entity labels and word tokens together to create a linear sentence. An example is shown in Figure 3. The generated linear sentences are input to a word generation network (such as an LSTM or BERT) to learn the distribution of words and tags. Given a sequence of tokens (w_1_,w_2_,...w_t_,...,w_N_) to the networks, where N denotes the length of the sequence, the networks learn the hidden states of each word in this sequence with equation 11:


h_t_ = Me_t_
**(11)**

where M denotes the learnable weight matrix in the word-generation networks and e_t_ denotes the embedding matrix of the input words. The word-generation networks learn to predict the tags of the next token in the sequence by maximizing the probability calculated by equation 12 in the process of training:



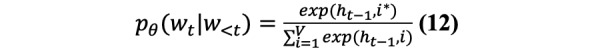



where *V* denotes the size of the vocabulary, i* denotes the index of the word w_t_ in the vocabulary, and h_t–1_,i* denotes the *i-th* element of h_t–1_. In this way, the objective function for obtaining the parameter θ is described in equation 13:



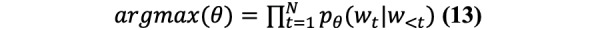



The paired token-label linear sentences promote learning by the networks of the context relationship between parts of speech, so the distribution of the generated synthetic data is closer to the original data, thereby introducing less noise during data augmentation. In addition, the generated synthetic data introduces more diversity to enhance the robustness of the model.

However, our originally collected data set may contain sentences that have fewer entities and more “O” tag words. According to equation 13, a DAGA heavily relies on contextual semantic information for sentence generation. Hence, only applying a DAGA to the originally collected data set for data augmentation may cause an entity sparsity issue, which is not conducive to a model for learning rich data features. To mitigate this, we introduced MR as another supplementary data augmentation method. For each labeled entity in a sentence, we formulated a binomial distribution to determine if the entity should be replaced. The formula outputs a probability *P*, and the entity is chosen for replacement by another entity from the training set that has the same entity type when *P*>.5. Otherwise, the entity remains in the original sentence. However, due to the small size of the originally collected data set, applying only MR for data augmentation easily generates duplicate data, which may cause oversampling in the training process, resulting in overfitting of the model. Therefore, we merged a DAGA and MR together to augment the data set.

## Results

### Data

The raw EHRs contain patient history, current illness, an admission summary, a daily record of the disease course, the diagnosis, treatment processes, and a discharge summary. The EHRs were all collected from local hospitals in Chongqing City, China. In this paper, we aim to identify protected information in the EHRs, such as the organization (ORG), location (LOC), dates (DAT), and personal names (PER), including the names of patients and doctors.

Manually annotating the raw data is a time-consuming and labor-intensive task, and the data are usually insufficient for disease-specific research, especially for rare diseases. Inspired by past research [28,29], we utilized a deep learning method for the raw data annotation. In this method, all the raw data are randomly split into 2 parts. The first part is called the “mini data set” (containing about 10% of all the raw data) and the other part is called the “formal data set.” We invited 2 professional clinicians to annotate all the PHI manually in the mini data set. Then, we fed the annotated mini data set to the base BERT with a CRF model to fine-tune it. Next, we switched the base BERT with the CRF model from a training mode to a test mode to predict the PHI in the formal data set. However, there may have been some incorrect predictions (also called bad cases) in the formal data set. Thus, we manually reviewed the predicted PHI in the formal data set and corrected the bad cases. In the end, we obtained a complete annotated data set with PHI labels. After that, private information, such as patient names, was replaced with random surrogates.

### Experiment Settings

We randomly split the raw annotated data set into a training set (denoted as data_raw_), an evaluation set, and a test set at a ratio of 6:2:2. Statistically, there were a total of 2707, 1424, 509, and 5046 labeled PER, ORG, LOC, and DAT entities, respectively. Our data augmentation method was applied to data_raw_ to create a new training set named the “hybrid augmented data set,” denoted as data_DAGA+MR_. For comparison, we separately applied a DAGA and MR to the data_raw_ to create 2 additional training sets, denoted as data_DAGA_ and data_MR_. The evaluation set was used for verifying performance in the training process and the test set was used for testing the performance of our proposed model and other baseline methods. Detailed statistical information on our hybrid augmented data set and the raw data set for each type of entity are shown in Table 1.

We retained the CRF module and replaced the feature extraction module of our model with other modules. These modules included 2 recurrent neural network (RNN)-based models, including BiLSTM [30], gated recurrent units (GRU) [31], and 7 BERT-based models, including base BERT [26], Chinese-BERT-wwm [32], Chinese-BERT-wwm-ext [32], Chinese-BERT-base [33], and Chinese-BERT-large [33], and as baselines, PCL-BERT [34] and PCL-BERT-wwm [34]. Detailed settings for each benchmark model are listed in Table 2. For the evaluation metrics, we used precision, recall, and the F1 score to evaluate the overall performance in the data sets, calculated according to equations (14) to (16), as follows:



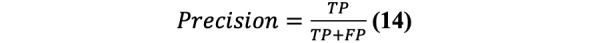





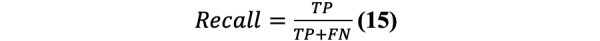





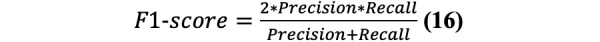



where TP, FP, and FN denote true positive number, false positive number, and false negative number, respectively. In practice, the experiments with the base BERT, Chinese-BERT-wwm, Chinese-BERT-wwm-ext, Chinese-BERT-base, Chinese-BERT-large, and TinyBERT models were conducted on a computer with an Intel Xeon central processing unit (CPU) (E5-2620, v3, 2.40 GHz) with 128 GB memory. The experiments with the GRU, BiLSTM, PCL-MedBERT, and PCL-MedBERT-wwm were conducted on an Nvidia RTX3090 graphics processing unit (GPU).

**Table 1:** Statistical information for the raw data and hybrid augmented data for each type of entity.

Entity types	Training set, n					Evaluation set (original), n		Test set (original), n
	Original	DAGA^a^	MR^b^	Total				
PER^c^	1448	4327	2892	8667	631		628	
LOC^d^	302	1384	589	2275	102		105	
ORG^e^	846	2188	1692	4726	275		303	
DAT^f^	3013	7412	6011	16,436	999		1034	
Total	5609	15,311	11,184	32,104	2007		2070	

^a^DAGA: data augmentation with a generation approach.

^b^MR: mention replacement.

^c^PER: personal name.

^d^LOC: location.

^e^ORG: organization name.

^f^DAT: date.

**Table 2:** Settings for each benchmark.

Models	Settings	Parameters, n	Description
Gated recurrent units	1 layer,^a^ 512 dims^b^	2,190,000	The parameters were randomly initialized.
BiLSTM^c^	1 layer, 512 dims	2,210,000	The parameters were randomly initialized.
Base BERT^d^	12 layers, 768 dims, 12 heads^e^	110,000,000	The base BERT was pretrained on the English Wikipedia corpus.
Chinese-BERT-wwm	12 layers, 768 dims, 12 heads	110,000,000	The base BERT was pretrained on the Chinese Wikipedia corpus with a whole word masking training strategy.
Chinese-BERT-wwm-ext	12 layers, 768 dims, 12 heads	110,000,000	The base BERT was pretrained on the Chinese Wikipedia corpus, news, and question-answer pairs with a whole word masking training strategy.
Chinese-BERT-base	12 layers, 768 dims, 12 heads	147,000,000	The base BERT was pretrained on the Chinese Wikipedia corpus with char, glyph, and pinyin embedding.
Chinese-BERT-large	24 layers, 1024 dims, 12 heads	374,000,000	The base-BERT-large model with more layers and larger dims was pretrained on the Chinese Wikipedia corpus using char, glyph, and pinyin embedding.
PCL-MedBERT	12 layers, 768 dims, 12 heads	110,000,000	A BERT model that was pretrained on the Chinese medicine corpus.
PCL-MedBERT-wwm	12 layers, 768 dims, 12 heads	110,000,000	A BERT model that was pretrained on the Chinese medicine corpus with whole word masking training.
TinyBERT	6 layers, 768 dims, 12 heads	67,000,000	A BERT distilled from the Chinese-BERT-wwm.

^a^Layer: transformer blocks.

^b^Dims: embedding dimensions.

^c^LSTM: long short-term memory.

^d^BERT: bidirectional encoder representations from transformers.

^e^Heads: attention heads.

### Experiment Results

The performance of our model compared with the baseline models on the test set is reported in Table 3. After fine-tuning data_raw_, base BERT obtained the best precision (98.55%), while PCL-MedBERT-wwm achieved the best recall (99.18%) and F1 score (98.8%). However, after fine-tuning the models on the hybrid augmented data set, our model obtained the best scores for precision (98.7%), recall (99.13%), and F1 score (98.91%), representing increases of 0.86% for precision, 0.53% for recall, and 0.69% for F1 score compared with data_raw_. Nevertheless, the other baseline models gained improved performance after fine-tuning on the hybrid augmented data set compared to data_raw_. Furthermore, the overall performance of the 2 RNN-based models was inferior to most of the BERT-based models, and the BiLSTM outperformed the GRU on precision, recall, and F1 score by 2.2%, 2.95%, and 2.58%, respectively, after training on data_raw_, and by 1.63%, 2.37%, and 2%, respectively, after training on the hybrid augmented data set.

It is worth noting that the performance of Chinese-BERT-base and Chinese-BERT-large were worse than the other BERT-based benchmark models after fine-tuning on data_raw_. The improvement of these 2 models surpassed the other models after fine-tuning on the augmented data set. Compared to fine-tuning on data_raw_, Chinese-BERT-base achieved increases of 13.94% for precision, 11.69% for recall, and 12.84% for F1 score, and Chinese-BERT-large achieved increases of 1.85% for precision, 0.87% for recall, and 1.36% for F1 score.

In order to further evaluate the effectiveness of our hybrid data augmentation method, we conducted an ablation study through fine-tuning each benchmark on data_DAGA_ and data_MR_. The results are shown in Table 4. Each metric of our model fine-tuned on either data_DAGA_ or data_MR_ performed better than when fine-tuned on data_raw_. The precision, recall, and F1 score improved 0.48%, 0.43%, and 0.46%, respectively, after fine-tuning our model on data_MR_, and improved 0.34%, 0.48%, and 0.38%, respectively, after fine-tuning on data_DAGA_. However, fine-tuning on a single augmented data set could not ensure that our model outperformed other baseline methods on each metric. Overall, the PCL-MedBERT-wwm obtained the best precision and F1 score after fine-tuning on data_MR_ and data_DAGA_.

It is worth noting that the results of some baseline benchmarks degraded after fine-tuning on data_MR_ or data_DAGA_. For example, after fine-tuning the models on data_MR_, the performance of PCL-MedBERT decreased 0.19% for precision, recall, and F1 score, and the performance of base BERT decreased 0.3%, 0.1%, and 0.2% for precision, recall, and F1 score, respectively. The situation was similar for Chinese-BERT-wwm-ext and Chinese-BERT-large. The performance of Chinese-BERT-wwm-ext decreased 0.29% for precision and 0.05% for F1 score, and the performance of Chinese-BERT-large decreased 0.47% for precision. Nevertheless, the performance of all the benchmark models improved after fine-tuning on our hybrid augmented data set, which proves the effectiveness of the proposed hybrid augmentation method.

We compared the performance on various entity types of our model after fine-tuning it on different data sets. As shown in Table 5, fine-tuning our model on either a single augmented data set or the hybrid augmented data set improved the performance for each entity type, which demonstrates the effectiveness of our proposed data augmentation strategy. It is worth noting that our model could not achieve the best performance for the PER and DAT entity types after fine-tuning on the hybrid augmented data set. For the DAT type, the best results were obtained after fine-tuning our model on data_MR_, with increases of 0.1% for precision, 0.29% for recall, and 0.19% for F1 score compared to the hybrid augmented data set. For the PER type, the best precision was obtained after fine-tuning our model on data_DAGA_; this was 0.16% higher than for data_DAGA+MR_.

To investigate the effect of data volume on our proposed model, we built 4 additional training sets with different data volume, denoted as 
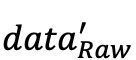
, 
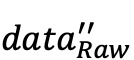
, 
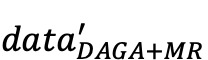
, and 
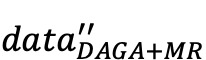
. These symbols and their corresponding meanings are listed in Table 6.

The results of our model after fine-tuning on the 4 additional training sets are shown in Table 7. From the table, we can observe that our model fine-tuned on 
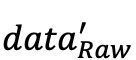
 only obtained performance of 91.33%, 95.26%, and 93.26% for precision, recall, and F1 score, respectively. When the volume of raw data increased to 50%, the performance improved greatly. Furthermore, the performance of our model fine-tuned on either 
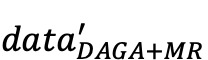
 or 
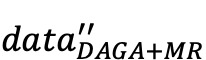
 was better than when fine-tuned on data_Raw_, 
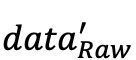
, or 
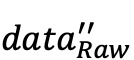
. Moreover, our model obtained better performance after fine-tuning on 
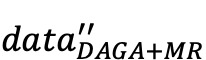
 than on 
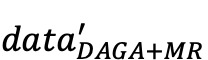
. The results also indicate that the less raw data we had, the more the performance of our model improved after fine-tuning on the hybrid augmented data set.

The time used by the different devices for all models that used the test set (including 1500 samples) was recorded for an efficiency evaluation. All the benchmarks ran a forwarded process on the test set; the results are shown in Table 8. Our model achieved the highest efficiency among all the BERT-based benchmarks: 158.22 seconds of CPU time and 62.39 seconds of GPU time. From the table, we can observe that the efficiency increase for CPU time was greater than for GPU time. The more limited were the computing resources, the greater was the efficiency improvement. These results show that our proposed method had higher efficiency with higher performance. Although the efficiency of the GRU and LSTM models was better than our model, the performance of these models for precision, recall, and F1 score was worse.

**Table 3:** Comparison of each benchmark model after fine-tuning on the raw data and the hybrid augmented data. Italics indicate the best performance.

Models	Data_raw_				Data_DAGA+MR_^a^		
	P,^b^ %	R,^c^ %	F1,^d^ %	P, %		R, %	F1, %
Gated recurrent units	94.92	93.04	93.97	95.9		95.02	95.46
BiLSTM^e^	97.12	95.99	96.55	97.53		97.39	97.46
Base BERT^f^	*98.55*	98.7	98.63	98.65		98.85	98.75
Chinese-BERT-wwm	98.35	98.5	98.43	98.5		98.90	98.7
Chinese-BERT-wwm-ext	98.4	98.5	98.45	98.65		98.90	98.78
Chinese-BERT-base	82.92	85.36	84.12	96.86		97.05	96.96
Chinese-BERT-large	95.42	95.7	95.56	97.27		96.57	96.92
PCL-MedBERT	98.37	99.08	98.72	98.36		98.79	98.58
PCL-MedBERT-wwm	98.42	*99.18*	*98.8*	98.46		98.89	98.67
Our model	97.84	98.6	98.22	*98.7*		*99.13*	*98.91*

^a^DAGA+MR: data augmentation with a generation approach and mention replacement.

^b^P: precision.

^c^R: recall.

^d^F1: F1 score.

^e^BiLSTM: bidirectional long short-term memory.

^f^BERT: bidirectional encoder representations from transformers.

**Table 4:** Ablation studies of each model fine-tuned on different data sets. Italics indicate the best performance.

Models	Data_raw_			Data_MR_^a^			Data_DAGA_^b^		
	P,^c^ %	R,^d^ %	F1,^e^ %	P, %	R, %	F1, %	P, %	R, %	F1, %
Gated recurrent units	94.92	93.04	93.97	95.68	94.2	94.94	94.64	94.59	94.61
BiLSTM^f^	97.12	95.99	96.55	97.72	97.15	97.43	97.14	96.86	97
Base BERT^g^	*98.55*	98.7	98.63	98.25	98.6	98.43	98.6	98.5	98.55
Chinese-BERT-wwm	98.35	98.5	98.43	98.5	98.7	98.6	98.45	98.7	98.58
Chinese-BERT-wwm-ext	98.4	98.5	98.45	98.11	98.7	98.4	98.8	98.9	98.85
Chinese-BERT-base	82.92	85.36	84.12	88.37	88.88	88.63	94.42	95.7	95.06
Chinese-BERT-large	95.42	95.7	95.56	94.95	96.42	95.68	97.53	97.25	97.39
PCL-MedBERT	98.37	99.08	98.72	98.18	98.89	98.53	98.7	*99.23*	98.96
PCL-MedBERT-wwm	98.42	*99.18*	*98.8*	*98.51*	98.99	*98.75*	*98.94*	99.13	*99.03*
Our model	97.84	98.6	98.22	98.32	*99.03*	98.68	98.18	99.08	98.6

^a^MR: mention replacement.

^b^DAGA: data augmentation with a generation approach.

^c^P: precision.

^d^R: recall.

^e^F1: F1 score.

^f^BiLSTM: bidirectional long short-term memory.

^g^BERT: bidirectional encoder representations from transformers.

**Table 5:** Performance comparison of our model on various entity types after fine-tuning our model with different data sets. Italics indicate the best performance.

Methods	PER^a^				LOC^b^				ORG^c^				DAT^d^		
	P,^e^ %	R,^f^ %	F1,^g^ %	P, %		R, %	F1, %	P, %		R, %	F1, %	P, %		R, %	F1, %
Data_raw_	99.21	99.52	99.36	96.15		95.24	95.69	97.06		98.02	97.54	97.42		98.55	97.98
Data_DAGA_^h^	99.37	*99.84*	99.6	95.28		96.19	95.73	96.43		98.02	97.22	98.27		99.23	98.75
Data_MR_^i^	99.36	99.36	99.36	94.44		97.14	95.77	96.1		97.69	96.89	*98.75*		*99.42*	*99.08*
Data_DAGA+MR_	*99.84*	99.68	*99.76*	*96.23*		*97.14*	*96.68*	*97.39*		*98.68*	*98.03*	98.65		99.13	98.89

^a^PER: personal name.

^b^LOC: location.

^c^ORG: organization name.

^d^DAT: date.

^e^P: precision.

^f^R: recall.

^g^F1: F1 score.

^h^DAGA: data augmentation with a generation approach.

^i^MR: mention replacement.

**Table 6:** Symbols and meanings of additionally built training sets.

Symbols	Meaning
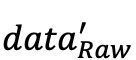	Randomly selected sample comprising 10% of data_raw_.
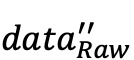	Randomly selected sample comprising 50% of data_raw_.
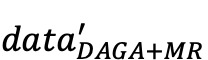 ^a,b^	Mixed data from 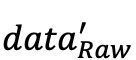 and the entire data set generated by DAGA and MR.
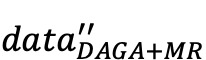	Mixed data from 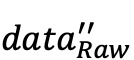 and randomly selected data generated by DAGA and MR.

^a^DAGA: data augmentation with a generation approach.

^b^MR: mention replacement.

**Table 7:** Results of TinyBERT after fine-tuning on different data volumes.

Data Volume	P,^a^%	R,^b^%	F1,^c^%
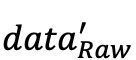	91.33	95.26	93.26
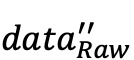	97.46	98.36	97.91
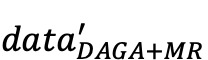 ^d,e^	98.13	98.89	98.51
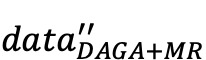	98.51	99.08	98.8

^a^P: precision.

^b^R: recall.

^c^F1: F1 score.

^d^DAGA: data augmentation with a generation approach.

^e^MR: mention replacement.

**Table 8:** Efficiency comparison of the benchmark models.

Models	CPU^a^ time, seconds	Difference vs our model, %	GPU^b^ time, seconds	Difference vs our model, %
Gated recurrent units	100.76	–36.31	56.45	–9.52
BiLSTM^c^	98.61	–37.68	54.94	–11.94
Base BERT^d^	262.81	39.8	78.02	20.03
Chinese-BERT-wwm	259.96	39.16	78.07	20.08
Chinese-BERT-wwm-ext	263.23	39.89	77.64	19.64
Chinese-BERT-base	220.93	28.38	76.28	18.21
Chinese-BERT-large	698.99	77.36	117.05	46.7
PCL-MedBERT	261.53	39.5	76.44	18.38
PCL-MedBERT-wwm	260.38	39.23	78.02	20.03
Our model	158.22	N/A^e^	62.39	N/A

^a^CPU: central processing unit.

^b^GPU: graphics processing unit.

^c^BiLSTM: bidirectional long short-term memory.

^d^BERT: bidirectional encoder representations from transformers.

^e^N/A: not applicable.

### Case Studies

**Figure 4:**
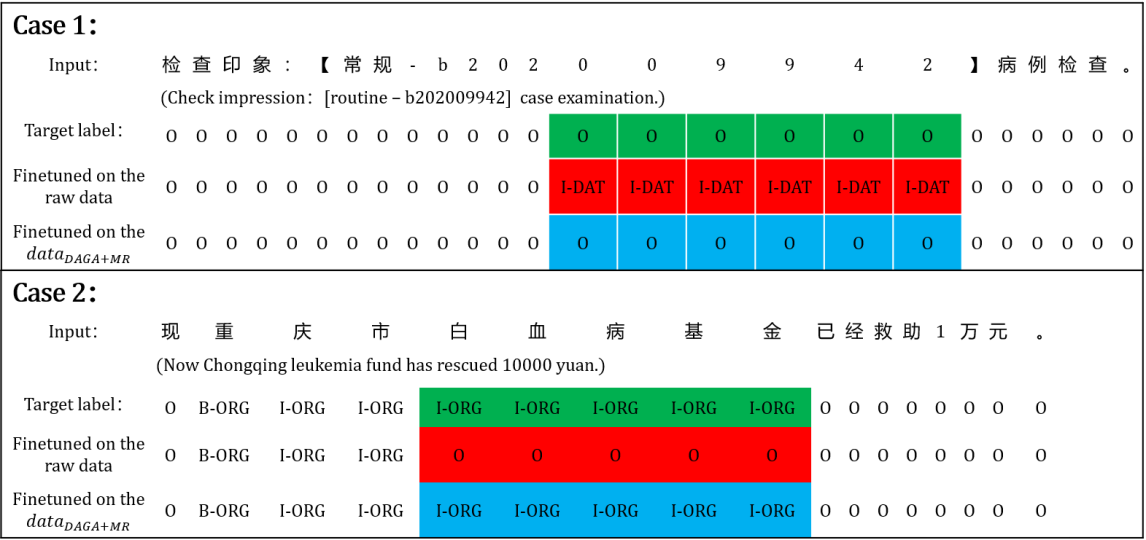
Examples of the results of fine-tuning our model on the hybrid augmented data set. DAT: date; ORG; organization name; DAGA: data augmentation with a generation approach; MR: mention replacement.

To visually verify the effectiveness of our proposed method, we used case studies as examples, as shown in Figure 4. In case 1, our model incorrectly classified the number “009942” from the “O” type as the DAT type after fine-tuning on the raw data. This was corrected after fine-tuning on our hybrid augmented data set. In case 2, the entity “白血病基金” (leukemia fund), which should have the ORG type, was not recognized when our model was fine-tuned on the raw data. However, our model was able to modify this result through context semantics after fine-tuning on the hybrid data set. These case studies demonstrate the effectiveness of the hybrid data augmentation method.

## Discussion

**Figure 5:**
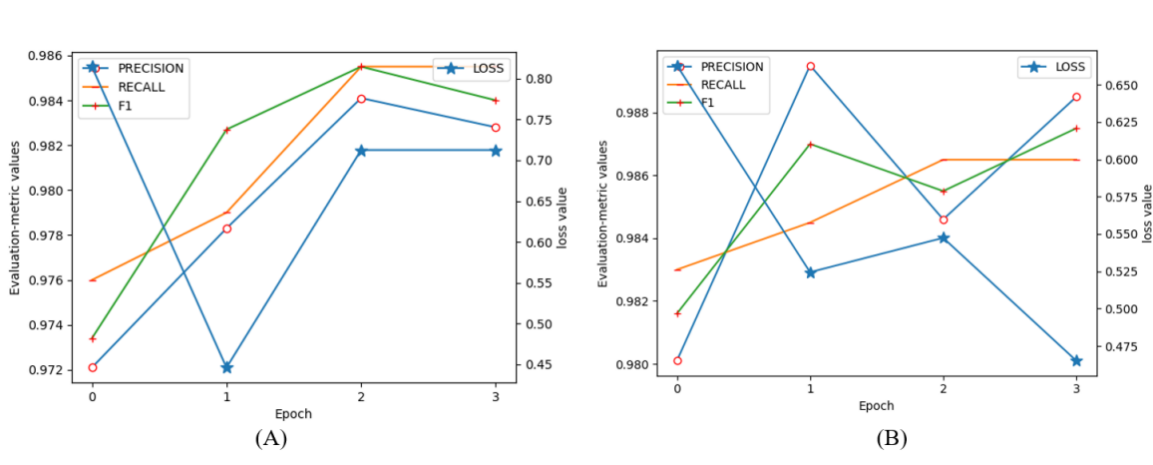
Training curves of our model on (A) the raw data set and (B) the hybrid augmented data set.

The main contributions of this paper are to (1) describe a new and efficient model that incorporates a TinyBERT and a CRF model to deidentify PHI in Chinese EHRs; (2) describe a hybrid data augmentation method utilizing a sentence generation strategy and an MR strategy for enhancing Chinese EHRs; and (3) report that our proposed method surpasses other baseline methods on both performance and efficiency. This could be for two possible reasons. First, the attention mechanism of TinyBERT and the optimal searching strategy of the CRF model ensured that our model learned the global features of texts well, and the lightweight parameters kept it from overfitting in the training process. Second, the DAGA generated more training data with more diversity and less noise for increasing the prior knowledge of data distribution for learning. The MR strategy randomly replaced entities in a sentence for learning representations of entities from diverse perspectives, which provided richer contextual information. The worse performance a model had after fine-tuning on raw data, the greater the performance improvement it could obtain after fine-tuning on the hybrid augmented data set. Additionally, the training curves of our model on data_raw_ and data_DAGA+MR_ are shown in Figure 5. This shows that our model quickly converged during training, which greatly reduced the training cost.

In addition, we performed an analysis to determine why the performance of some baseline methods degraded after fine-tuning on data_raw_ or data_DAGA_. We found that, on the one hand, there may have been disadvantages arising from the data sparsity of data_DAGA_, which hampered the ability of the models to focus on useful contextual semantic information in sentences, impairing feature extraction. On the other hand, applying the MR strategy to the raw data set tended to generate duplicate data, which could have resulted in overfitting in the training process. These 2 shortcomings had a greater impact on the Chinese-BERT-large model, because that model has more transformers and parameters, and is therefore more sensitive to data disturbances [25]. However, the hybrid augmented data could not ensure that our model improved its performance on each type of entity, although the performance on the overall test set was still improved. Moreover, the pretraining data set had a great impact on the downstream tasks. Though the base BERT was pretrained on the English corpus, it obtained much better performance than Chinese-BERT-base. Chinese-BERT-base and Chinese-BERT-large were pretrained with Chinese word information including words, glyphs, and pinyin information. In our experiments, we solely fine-tuned these models with word information, like the other BERT-based models, and found that this led to heavy performance degradation on data_raw_.

The input of our proposed model is structured data, which needs to be correctly prepared from the raw collected data. Although we employed a BERT model to improve the efficiency of the annotating process, the generalizability of this method to EHRs in different languages has not been proved. Furthermore, the location information could have correlations with disease type, although we did not specifically evaluate the influence of PHI deidentification on clinical data mining in this paper.

This paper proposes an efficient and effective model that integrates a TinyBERT and a CRF model for the task of deidentifying PHI in Chinese EHRs. This model relieves the high dependency on computing resources of previous models and improves the efficiency of the task. To overcome the limitation of insufficient annotated data, we propose a hybrid data augmentation method, which uses a generation approach and an MR strategy to create a new data set for fine-tuning the model. Our experimental results demonstrate that the performance of our model was greater than baseline models and also had the highest efficiency of all the experimental benchmark models.

## Abbreviations

BERT: bidirectional encoder representations from transformers
CPU: central processing unit
CRF: conditional random field
DAGA: data augmentation with a generation approach
DAT: date
EHR: electronic health record
GPU: graphics processing unit
GRU: gated recurrent units
KD: knowledge distillation
LOC: location
LSTM: long short-term memory
MR: mention replacement
NER: named entity recognition
ORG: organization name
PER: personal name
PHI: protected health information
RNN: recurrent neural network

## Data Availability

The data sets used and analyzed during the current study are available from the first author upon reasonable request.
